# A platinum promoted Ag/SBA-15 catalyst effective in selective oxidation of methanol – design and surface characterization†

**DOI:** 10.1039/d0ra01562h

**Published:** 2020-04-09

**Authors:** Joanna Wisniewska, Izabela Dziedzic, Maria Ziolek

**Affiliations:** Faculty of Chemistry, Adam Mickiewicz University in Poznań Uniwersytetu Poznańskiego 8 61-614 Poznań Poland jc62431@amu.edu.pl +48 61 8291794

## Abstract

The aim of this study was better understanding of surface properties of bimetallic (silver–platinum) catalysts and to verify if a very small addition of platinum (*ca.* 0.05 wt%) to silver (*ca.* 2.0 wt%) loaded on ordered mesoporous silica, SBA-15, would improve the catalytic properties of bimetallic Ag–Pt materials in selective oxidation of methanol to methyl formate. Ag–Pt catalysts were prepared by one-step and step-by-step procedures and the final Ag/Pt molar ratio in the respective samples was equal to 86 and 63. The catalysts were characterized after calcination and different activation treatments (in Ar and O_2_). X-ray diffraction, UV-vis and XP spectroscopy confirmed the lack of Ag–Pt alloy crystallites in the samples and also evidenced a higher resistance of silver oxide species to reduction upon activation in Ar flow in the presence of platinum promoter interacting with silver species. Methanol oxidation over the samples activated in Ar flow and in oxidizing flow (O_2_ + Ar) helped identify the role of each component in the bimetallic Ag–Pt catalyst in terms of activity and selectivity in the oxidation of methanol to methyl formate. A highly active bimetallic Pt/Ag/SBA-15 catalyst, selective to methyl formate and stable in methanol oxidation was constructed.

## Abstract 2

The aim of this study was better understanding of surface properties of Ag–Pt catalysts and to verify if addition of platinum promoter to silver loaded on SBA-15, would improve the catalytic properties of Ag–Pt materials in selective oxidation of methanol to methyl formate.

## Graphical abstract

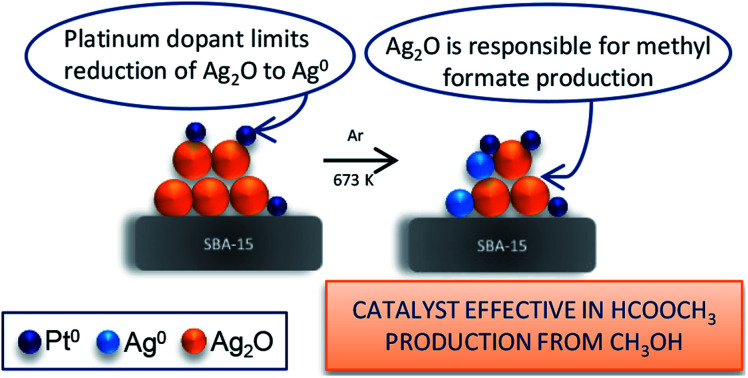

## 1. Introduction

Catalyst surface engineering is important in the construction of catalysts intended for desired processes. In this work the focus is on the design and study of surface properties of bimetallic (silver–platinum) materials based on ordered mesoporous silica (SBA-15) proposed for the selective oxidation of methanol to methyl formate.

Catalytic methanol oxidation to formaldehyde is applied in industry with the use of silver or iron–molybdenum catalysts.^1^ The application of both catalysts requires different reaction conditions. The first, silver catalyst works at higher temperatures (823–1023 K) and higher concentration of methanol in the reagents mixture, whereas the second catalyst works in the range 623–723 K in lower methanol concentration. It is well known that methanol oxidation is also an attractive process for production of other chemicals like methyl formate or dimethoxy methane.^2,3^ Both are commercially needed.

Methyl formate is a useful organic chemical and can be used as an intermediate for the preparation of a wide range of other chemicals like formic acid, higher carboxylic acids such as acetic acid and propionic acid and their esters. It is also used in cereal and tobacco crops as a fumigant, in the cellulose industry as a solvent, in foundries in the process of curing resins, in the curing of phenol esters. Production of methyl formate from methanol is desirable because methanol is a readily available starting material and can be obtained either from natural gas or from synthesis gas also generated from biomass conversion.^4–6^

One of the old patents^7^ presented an invention that described a process for the preparation of methyl formate from methanol in which methanol was contacted at elevated temperature with a platinum group metal catalyst in the absence of an added hydrogen acceptor. These metals were supported on inert solids, among others on silica, having hydroxyl groups capable of reacting with the platinum group compound. The preferable temperature range for this reaction was 413–453 K. The oxidation of methanol vapor over platinum catalysts was studied by many research groups.^8–11^ Latest works focused on the effects of platinum nanoparticle size and metal/support interactions. It has been found out^11–13^ that Pt cluster diameter influences the rate and selectivity of the reaction. Larger Pt clusters contain coordinatively more saturated surface Pt sites than smaller Pt clusters. It results in a weaker binding to chemisorbed oxygen and methanol derived intermediates. The preoxidized platinum catalyst Pt/Al_2_O_3_, in which a larger degree of oxidation of platinum was observed, was more active than the prereduced catalyst. The differences in reactivity were linked to the formation and stabilization of distinct active oxide species during the pretreatment.^9^

Inspired by metallic catalysts for the oxidation of methanol to formaldehyde and methyl formate we have combined both metals, silver and platinum loaded on silica supports as catalysts for selective methanol oxidation.^14–16^ Our previous studies^14^ have been focused on finding the optimal conditions of alloying silver and platinum loaded on different silicas and studying of their properties in methanol oxidation performed in the gas phase. We have been successful in preparation of samples containing Ag–Pt alloy nanoparticles. However, the alloy species were not very stable upon activation conditions which always takes place before oxidation processes.^14,17^ In the most of examined bimetallic Ag–Pt samples the activation in argon flow caused total or partial segregation of platinum and silver on the surface of silica support. It resulted in a decrease in activity and/or selectivity in methanol oxidation. From among the studied samples the only one in which the alloy structure was maintained was the catalyst based on mesoporous cellular foam (MCF) containing silver & platinum (Ag/Pt molar ratio = 4.2). This sample displayed 100% conversion of methanol with 73% selectivity to methyl formate at a relatively low temperature, 423 K. However, this catalyst not only showed decreased activity after a few hours of the reaction, but also changed selectivity and increased the total oxidation of methanol. The reason was the following segregation of alloy phase and agglomeration of single metal particles during the reaction performed under the applied conditions. After segregation of alloy components, both silver and platinum species acted as separate phases active mainly in total oxidation of methanol to CO_2_. Such instability of the bimetallic silver & platinum catalysts provoked us to look for a different composition of Ag & Pt catalysts. The focus was on such a composition that would contain significantly less platinum than silver, because separated platinum species (formed during methanol oxidation as a result of alloy segregation) mainly activated the total oxidation of methanol.

The research hypothesis in this work was that a very small addition of platinum to silver loaded on ordered mesoporous silica, SBA-15, would be able to improve selectivity of the catalyst into methanol oxidation to methyl formate and that a platinum promoter would provide higher stability of the catalyst during methanol oxidation and thus allows getting stable methyl formate production. To check this hypothesis, SBA-15 material was modified with 2.0 wt% of silver and 0.1 wt% of platinum applying two different procedures, one-step and step-by-step modification, first silver and next platinum loading. The results of methanol oxidation on so-prepared samples appeared to be promising for selective formation of methyl formate.

## 2. Experimental

### 2.1. Synthesis and functionalization of SBA-15 support with 3-mercaptopropyltrimethoxysilane (MPTMS)

The SBA-15 support was prepared according to the procedure described in ref. 18 and 19. It was grafted with 3-mercaptopropyltrimethoxysilane (MPTMS) (95%, Sigma-Aldrich) to functionalize its surface before modification with metals, according to our previous work^14^ (S1-ESI†). The functionalized material is marked as SBA-SH.

### 2.2. Modification of functionalized support with platinum and/or silver

Bimetallic catalysts containing silver and platinum were prepared according to two approaches: one-step or step-by-step modification of functionalized support with metal precursors. The assumed metals loading in both cases was 2.0 wt% for silver and 0.1 wt% for platinum.

#### 2.2.1 One-step method – simultaneous incorporation of two metals

The preparation procedure of the bimetallic Ag–Pt system was as follows: 25 mL of water solution of silver nitrate (AgNO_3_, ≥99.8%, Sigma-Aldrich) and 25 mL of water solution of hexachloroplatinate(iv) hydrate (H_2_PtCl_6_, 38.84%, Johnson Matthey) were prepared separately and then mixed for 5 min in one flask. Then the functionalized support was stirred for 4 h together with the prepared solution at room temperature. The solid was recovered by filtration and washed with 80 mL of distilled water. The recovered material was stirred at room temperature with 60 mL of 0.1 M sodium borohydride (NaBH_4_, >99.8%, Sigma-Aldrich) solution. After 40 min, the solid was recovered by filtration, washed with 80 mL of distilled water. The material was dried at 333 K for 12 h and calcined in air at 773 K for 4 h. The calcination step was applied for removal of organosilane (MPTMS) which was used for functionalization of silica before anchoring of metal ions. The prepared sample was labelled as AgPt/S, where S stands for SBA-15.

#### 2.2.2 Step-by-step method – first silver next platinum anchoring

The preparation procedure was as follows: the functionalized material SBA-SH was stirred for 2 h at room temperature in 25 mL of water solution of silver nitrate. After filtration and washing with 80 mL of distilled water the recovered solid (R) was divided into two parts, R1 and R2. R1 was stirred at room temperature with 60 mL of 0.1 M NaBH_4_ solution for 40 min to obtain the reduced monometallic sample. The solid was recovered by filtration, washed with 80 mL of distilled water. Then it was dried at 373 K for 12 h and calcined at 773 K for 4 h. In this way the material marked as Ag/S was obtained, where S stands for SBA-15.

To prepare the bimetallic material, the second part of the recovered product (R2) was moved into the beaker and stirred for 2 h at room temperature in 25 mL of water solution of hexachloroplatinate(iv) hydrate H_2_PtCl_6_. The next steps were the same as above described for monometallic sample. Bimetallic material was labelled as Pt/Ag/S where S stands for SBA-15.

Monometallic platinum catalyst Pt/S was prepared in the same way as Ag/S with the use of hexachloroplatinate(iv) hydrate H_2_PtCl_6_ as platinum precursor, instead of silver nitrate.

### 2.3. Samples characterization

The materials prepared were characterized by using inductively coupled plasma optical emission spectrometry (ICP-OES), X-ray diffraction (XRD), N_2_-adsorption/desorption isotherms, Transmission Electron Microscopy (TEM), ultraviolet-visible (UV-vis) spectroscopy, and X-ray photoelectron spectroscopy (XPS). All these techniques are described in details in the ESI (S2†), according to our previous papers.^14,15^

### 2.4. Methanol oxidation

The catalytic reaction was performed in a fixed-bed flow reactor, as described in our previous works.^14,15^ A portion of 0.04 g of granulated catalyst of the size fraction of 0.5 < *Φ* < 1 mm was placed into a tubular reactor (diameter *Φ* = 5 mm, length *l* = 70 mm). The height of the catalyst bed was 6–7 mm. The temperature was controlled by a thermocouple located in the catalyst bed. The samples were activated in argon flow (40 mL min^−1^) or in oxidizing flow (10 mL min^−1^ O_2_ + 30 mL min^−1^ Ar) at 673 K for 2 h (with a ramp of 15 K min^−1^). Then, the temperature was decreased to that of the reaction. The reactant mixture of Ar/O_2_/CH_3_OH (88/8/4 mol%) was supplied at the rate of 40 mL min^−1^. The external (inspected by tests with the use of different volumes of catalysts and constant granular size of catalysts and contact time) and internal (inspected by tests with the use of different granular sizes of catalyst and constant volume of catalyst and contact time) diffusion do not limit the reaction rate in these conditions. Methanol (Chempur, Poland) was introduced to the flow reactor by bubbling argon gas through a glass saturator filled with methanol. The reactor effluent was analyzed using two online gas chromatographs. One gas chromatograph, GC 8000 Top, was equipped with a capillary column of DB-1, operated at 313 K, and a FID detector applied for analyses of organic compounds, while the other GC containing Porapak Q and 5A molecular sieves columns (used for analyses of O_2_, CO_2_, CO, H_2_O, and CH_3_OH) had a TCD detector. The columns in the second chromatograph with TCD were heated according to the following program: 5 min at 358 K, temperature increase to 408 K (heating rate 5 K min^−1^), 4 min at 408 K, cooling to 358 K (for the automatic injection onto the column with 5A), 10 min at 358 K, temperature increase to 408 K (heating rate 10 K min^−1^), and 11 min at 408 K. Argon was applied as a carrier gas. The outlet stream line from the reactor to the gas chromatograph was heated at about 373 K to avoid condensation of the reaction products. The selectivity, *S*_i_, was calculated as the molar concentration of the indicated product (i) divided by the sum of the concentrations of all products detected, *S*_i_ = *c*_i_/∑*c* × 100%. The following products detected by FID and TCD detectors were analyzed: methanol, formaldehyde, methyl formate, dimethoxymethane, dimethyl ether, and carbon dioxide. The product distribution was illustrated by selectivities.

## 3. Results and discussion

### 3.1. Composition and structure of catalysts

To confirm typical structure of SBA-15, pure support and silica modified with metals were examined by X-ray diffraction in small-angles range. Fig. S1-ESI† shows the XRD patterns of the prepared support and metallic samples. Three distinct peaks assigned to the (100), (110) and (200) reflections were observed in the 2*θ* ranges between 0.5° and 2°, which are characteristic of SBA-15 with *p*6*mm* 2D-hexagonal symmetry. Thus, modification of mesoporous silica did not change the mesoporous structure of the support. Textural parameters calculated from N_2_ adsorption/desorption isotherms (Fig. S2-ESI†) of all samples prepared are summarized in Table 1. The surface area reached 785 m^2^ g^−1^ for pure support and *ca.* 400–500 m^2^ g^−1^ for the support modified with metals. There are no significant differences in the average pore diameter and total pore volume of prepared monometallic and bimetallic catalysts. Metal modification of silica support firstly functionalized by MPTMS led to a decrease in surface area, pore diameter and pore volume after introduction of modifiers. The chemical composition of the samples was estimated on the basis of ICP-OES and the results are collected in Table 1. The assumed metals loading was equal to 2.0 wt% for silver and 0.1 wt% for platinum. The modification efficiency reached 100% for monometallic silver sample and 87–95% for silver in bimetallic materials. The efficiency of platinum introduction was *ca.* 50% for all samples. The real Ag/Pt molar ratio for the sample prepared in the one-step modification was equal to 86 (AgPt/S), whereas for the sample prepared by the step-by-step method it was equal to 63 (Pt/Ag/S). These Ag/Pt molar ratios are much higher than those required for the formation of Ag–Pt alloy nanoparticles.^20^ In the one-pot modification the initial solution contained 20 times lower amount of platinum ions than silver. It is possible that much higher amount of Ag^+^ ions interacting with thiol groups (SH from MPTMS – functionalization agent) limited the access of PtCl_6_^−^ to anchoring sites. A kind of competition between Pt and Ag sources occurred. In contrast, during the step-by-step modification procedure, silver species (Ag^+^ ions from silver nitrate) added firstly were strongly held on SH groups. In such a form they provided good adsorption sites for anchoring of PtCl_6_^−^. It can be a reason for a higher amount of platinum anchored in this procedure.

**Table 1:** The chemical composition and texture parameters of studied samples

Catalyst^a^	Pt [wt%]	Ag [wt%]	Ag/Pt molar ratio -assumed/real	BET surface area (m^2^ g^−1^)	Total pore volume, DFT (cm^3^ g^−1^)	Average pore diameter, DFT (nm)
S	—	—	—	785	0.94	10.1
Pt/S	0.05			532	0.68	9.4
Ag/S	—	2.00	—	433	0.65	9.8
AgPt/S	0.04	1.90	36.0/85.9	457	0.69	9.4
Pt/Ag/S	0.05	1.74	36.0/63.0	416	0.67	9.3
0.5Pt2Ag/M^b^	0.50	1.1	7.2/4.2	—	—	—

a S stands for SBA-15 support.

b From our previous work,^8^ M stands for MCF support.

### 3.2. State of silver and platinum

#### 3.2.1 Catalysts after calcination

**Fig. 1:**
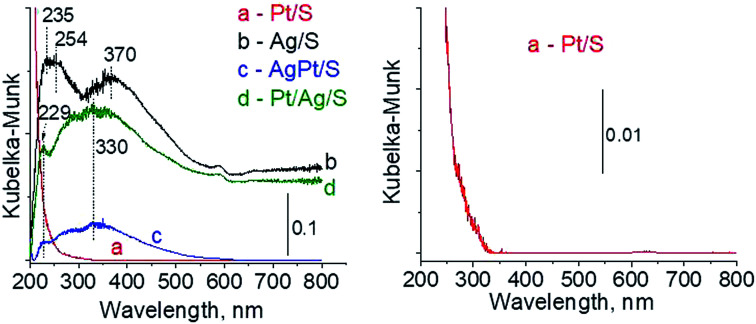
UV-vis spectra of the samples calcined at 773 K for 4 h.

The types of active species in calcined samples were identified firstly from UV-vis spectra (Fig. 1). According to literature the surface plasmon resonance (SPR) band of spherical metallic silver nanoparticles appears at *ca.* 400 nm,^21–23^ whereas the band at *ca.* 230–260 nm comes from 4d^10^/4d^9^s^1^ transition characteristic of well-dispersed Ag^+^ cations and/or small Ag_*n*_^*δ*+^ clusters.^24–26^ The spectra of Ag/S display two broad bands: at *ca.* 245 nm and *ca.* 370 nm. The first one seems to have two components at *ca.* 235 nm and *ca.* 254 nm, both assigned to silver cations in silver oxide. The second one is characteristic of metallic silver. All of them are slightly blue-shifted in comparison with literature data but it can be caused by many factors such as interaction with the support, or size and shape of particles. Although the spectrum of monometallic platinum sample is shown additionally in expanded scale (because of a very low content of platinum modifier) (Fig. 1) it still does not show any UV-vis bands. The spectra of bimetallic samples AgPt/S and Pt/Ag/S display a band at 229 nm from Ag^+^ cations.^24–26^ There is also a broad band with the center at *ca.* 330 nm. It can cover three bands: from metallic silver (*ca.* 400 nm), from silver Ag_*n*_^*δ*+^ clusters (*ca.* 260 nm) and from cationic platinum species Pt^4+^ (*ca.* 270 nm) or Pt^2+^ (*ca.* 380 and 417 nm). Thus, on the basis of UV-vis spectroscopy only it is not possible to unambiguously identify the metal species present in bimetallic AgPt/S and Pt/Ag/S samples.

**Fig. 2:**
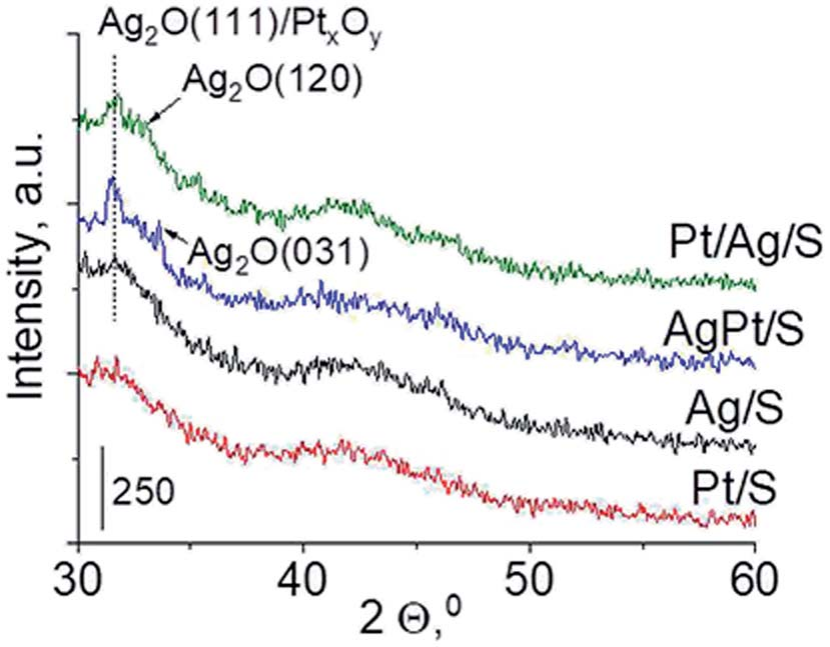
Wide-angle XRD patterns of the samples calcined at 773 K for 4 h.

Another technique which allows getting information on the metals phases present in the samples is X-ray diffraction. XRD patterns in the wide-angle range were examined to observe the presence of metal and/or metal oxide crystallites of modifiers. As shown in Fig. 2 the only reflections visible in XRD patterns are located at 2*θ* ≈ 31.7°, 2*θ* ≈ 33.0° and 2*θ* ≈ 33.7°. They come from Ag_2_O (111), (120) and (031) planes,^27^ respectively. The intensity of these reflections is higher for bimetallic samples, which suggests larger particles in these samples. It is worth mentioning that X-ray diffraction has some limitations – it permits detecting particles with diameters bigger than 1.5 nm and if their concentration is sufficiently high. Thus the lack of any reflection in XRD pattern of Pt/S can be caused by high dispersion of platinum particles or rather by too low content of this metal (0.05 wt%).

#### 3.2.2 Catalysts after activation at 673 K in argon flow (40 mL min^−1^ at 673 K)

**Fig. 3:**
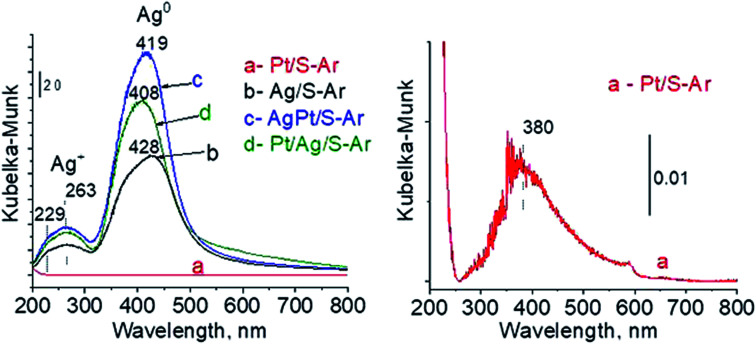
UV-vis spectra of the samples activated at 673 K in argon flow (40 mL min^−1^) for 2 h.

**Fig. 4:**
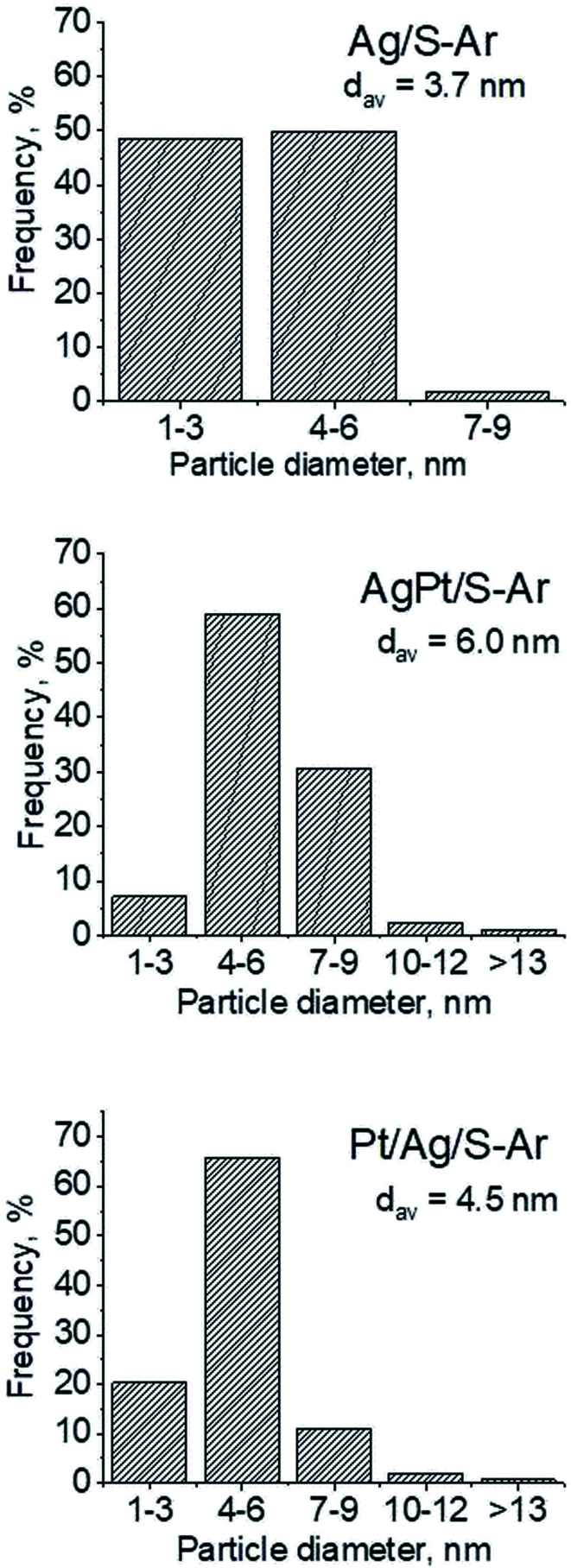
Metal particle sizes distribution on the support after activation at 673 K in Ar flow (40 mL min^−1^) for 2 h (basing on TEM images and measuring of 250 crystallites in each sample).

**Fig. 5:**
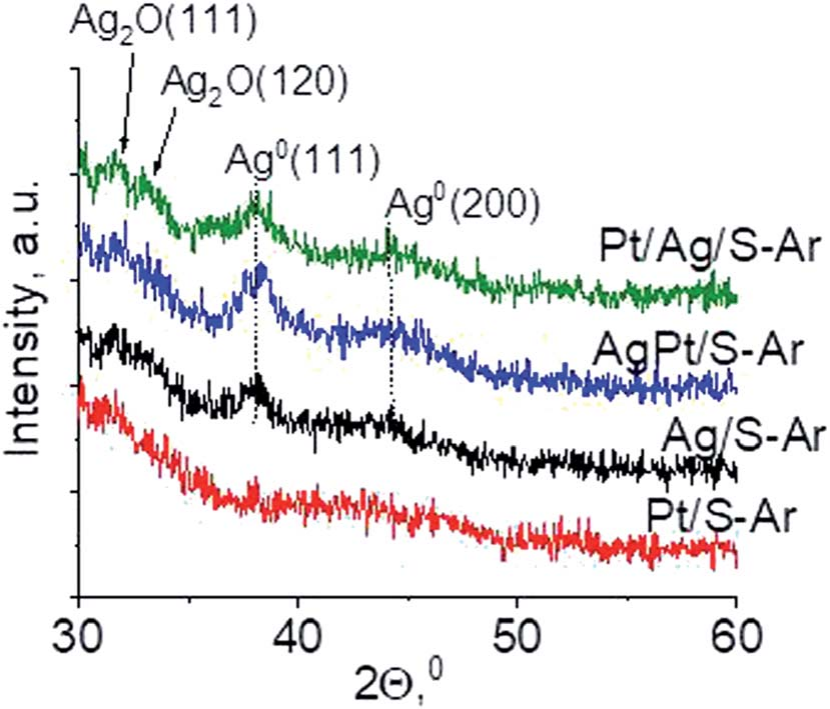
Wide-angle XRD patterns of the samples activated at 673 K in argon flow (40 mL min^−1^) for 2 h.

**Fig. 6:**
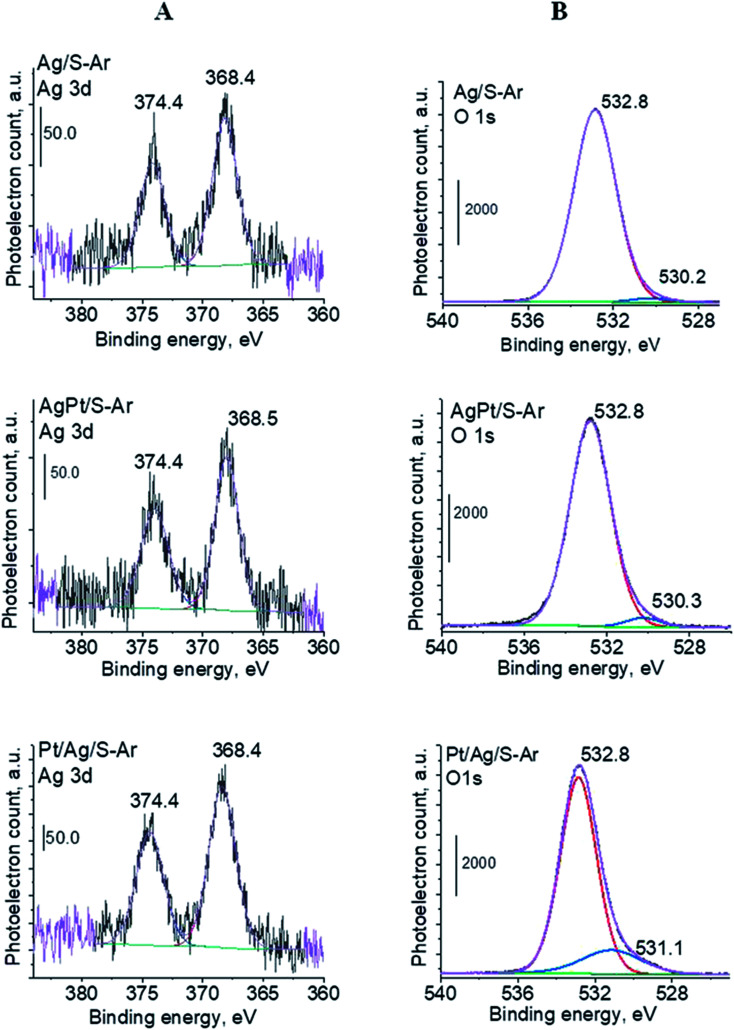
XPS spectra (Ag 3d (A) and O 1s regions (B)) for Ag/S, AgPt/S and Pt/Ag/S catalysts activated at 673 K in argon flow (40 mL min^−1^) for 2 h.

In our previous studies^17^ it has been shown that characterization of catalysts after activation (673 K in argon flow) is extremely important because the samples are activated before a catalytic reaction and their surface properties can be changed. High mobility of silver species as well as its susceptibility to changes in oxidation state was the reason for segregation of Pt–Ag alloy reported in ref. 14. Thus, this time we also turned our attention to the states of metal modifiers not only after calcination but mainly after activation which took place before methanol oxidation. The UV-vis spectra of the studied catalysts activated in argon flow at 673 K are collected in Fig. 3. All spectra display a well separated band located at *ca.* 400 nm coming from metallic silver Ag^0^.^23^ As expected, the activation in inert gas caused the reduction of silver oxide species to metallic form. The reduction of silver cationic species upon activation was not total, as the broad band characteristic of these species did not disappear completely. This band consists of two peaks, one at *ca.* 229 nm and the other at *ca.* 263 nm, both assigned to silver cations.^24–26^ The position of surface plasmon resonance band of metallic silver is worth of discussion. Monometallic silver sample exhibits this band at 428 nm, whereas a blue shift (to 419 nm for AgPt/S–Ar and 408 nm for Pt/Ag/S–Ar) is visible for bimetallic materials. It is clear that addition of platinum to silver changes the band position indicating a synergistic effect between metallic silver and platinum species. This effect was larger for bimetallic catalysts prepared by the step-by-step method because platinum introduced after silver loading on the support could partially covered silver species. The observed blue shift of SPR band could originate from a decrease in metal particle size. However, the analysis of TEM images discussed below excludes such a possibility. Thus, the blue shift comes from interactions between silver phase and platinum promoter. Careful look at the spectra of the platinum sample (Pt/S–Ar) reveals some changes in the spectra after activation of the sample in argon flow. The low-intensive broad band with the maximum at *ca.* 380 nm appeared, indicating the presence of partially reduced Pt^2+^ species.^28^ It suggests that upon activation Pt^4+^ cations accept electrons and Pt^2+^ is formed. It may limits reduction of silver oxide species. Transmission electron microscopy images shown in Fig. S3-ESI† allow determination of metal particle size distribution in the samples activated in argon flow (Fig. 4). It is well visible that in bimetallic samples the average particle size of metal species significantly increased in comparison with the size of silver nanoparticles (NPs) in monometallic sample. It is also important to stress that in monometallic silver containing catalyst the contribution of very small Ag NPs is almost 50%, whereas the share of these NPs in bimetallic samples is significantly lower. The XRD patterns of activated samples (Fig. 5) confirmed the results from UV-vis spectroscopy and TEM analysis. Two additional reflections at 2*θ* ≈ 38.1° and 2*θ* ≈ 44.1° confirmed the presence of metallic silver crystallites.^29,30^ No reflections which could be assigned to bimetallic Ag–Pt alloy-like crystallites^14^ appeared in the XRD patterns. The appearance of reflections from Ag^0^ is correlated with reduced intensity of the reflections at 2*θ* ≈ 33.7° and 2*θ* ≈ 31.7° coming from silver oxide. In the XRD pattern of the monometallic sample Ag/S–Ar the reflections from oxide species disappeared totally, which suggests that the reduction of silver oxide was deeper in this sample. It means that platinum species partially stabilized silver oxide species. To get more information about the oxidation states of metals on the silica surface, the XPS spectra of the samples after activation were recorded. In the spectrum of Ag/S–Ar (Fig. 6A), two well defined bands assigned to metallic Ag 3d_5/2_ (binding energy (BE) 368.4 eV) and Ag 3d_3/2_ (BE 374.4 eV) indicated that silver species on the surface occurred as Ag^0^ species.^31–33^ The XPS spectra of the bimetallic catalysts display the Ag 3d bands in exactly the same positions as those of the monometallic sample. On the basis of literature data^31–33^ and our own studies,^14^ it has been concluded that the ranges of binding energies of Ag 3d_5/2_ reported for Ag^0^, Ag_2_O and AgO overlapped. Thus, to check if there are some metal oxides on silica surface the XP spectra in O 1s region were deconvoluted and examined (Fig. 6B). All materials display an intense band corresponding to Si–O–Si bonds in silica structure (binding energy 532.8 eV). In the spectra of Ag/S–Ar and AgPt/S–Ar the other band centered at *ca.* 530.2 eV is also visible. It comes from the oxygen in Ag–O–Ag bonds.^34^ The band at 531.9 eV visible in Pt/Ag/S–Ar spectrum can be assigned to the oxygen from surface hydroxyl groups^35^ or the subsurface oxygen in silver species.^34^ It is worth to note that the band coming from Ag–O–Ag bonds is much larger in bimetallic catalysts compared to Ag/S–Ar catalyst. These results are in accordance with XRD studies of activated samples and confirm that the reduction of silver upon activation was inhibited if platinum was added to the sample.

To sum up: on the basis of X-ray diffraction, UV-visible and XP spectroscopy of catalysts activated in argon flow we can conclude that silver in all samples was present in both forms: Ag^0^ and Ag_2_O, however the reduction of silver oxide was deeper in monometallic silver catalysts Ag/S. It is not possible to say explicitly that platinum was in the metallic or oxide form because of very low concentration of platinum in materials (0.05 wt%), which did not allow their detection by the used techniques. The only form which was detected by UV-vis spectroscopy was Pt^2+^ species. In the activated samples there was no silver–platinum nanoalloy, but the blue shift in the UV-vis spectra proves interactions between platinum promoter and silver phase.

#### 3.2.3 Catalysts after activation at 673 K in oxidizing mixture (10 mL min^−1^ of O_2_ + 30 mL min^−1^ of Ar)

**Fig. 7:**
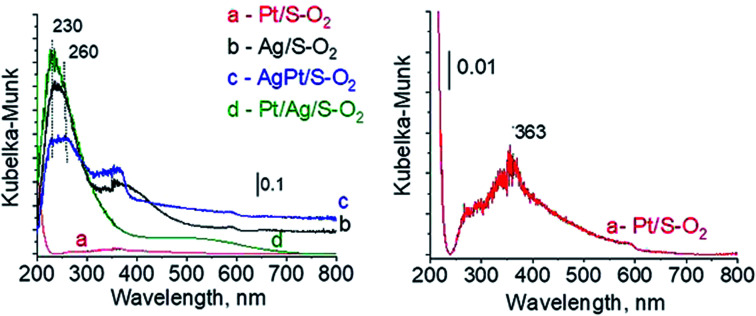
UV-vis spectra of the catalysts activated at 673 K in oxidative mixture flow (10 mL min^−1^ O_2_ + 30 mL min^−1^ Ar) for 2 h.

**Fig. 8:**
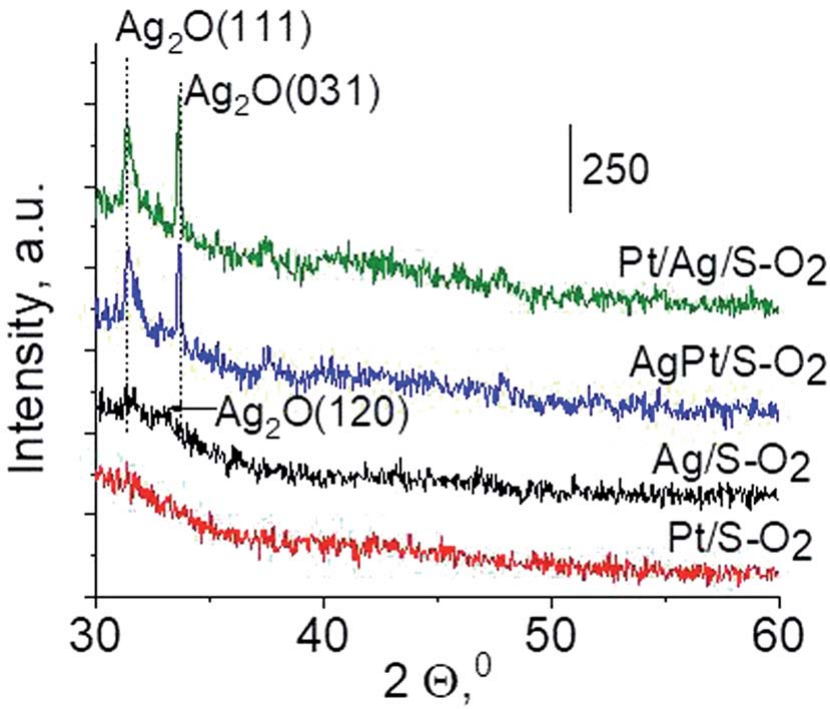
Wide-angle XRD patterns of the samples activated at 673 K in oxidative mixture flow (10 mL min^−1^ O_2_ + 30 mL min^−1^ Ar) for 2 h.

For deeper understanding of cationic silver/silver oxide role in methanol oxidation (results shown in the next section) the materials were studied after treatment in oxidizing mixture. As seen in Fig. 7 the UV-vis spectra of activated Ag/S–O_2_, AgPt/S–O_2_ and Pt/Ag/S–O_2_ samples display the main band, which covers both peaks at *ca.* 230 nm and *ca.* 260 nm coming from cationic silver species.^24,25^ Two samples (Ag/S–O_2_ and AgPt/S–O_2_) additionally display a less intense band at *ca.* 370 nm from small metallic silver clusters Ag_*n*_. The spectrum of Pt/Ag/S–O_2_ synthesized by the step-by-step method differs from the other ones because it shows only one band characteristic of cationic silver. Interestingly, the spectrum of Pt/S–O_2_ is similar to that of Pt/S–Ar (Fig. 7) and shows a broad band with a maximum at 363 nm suggesting the presence of cationic platinum species Pt^2+^, probably from PtO particles. The presence of silver oxide/cationic silver clusters in activated samples was confirmed by X-ray Diffraction (Fig. 8). From XRD patterns it is clearly seen that silver oxide particles are bigger in bimetallic catalysts (very intense reflections) than in Ag/S–O_2_ sample. It indicates that addition of very small amounts of platinum (0.05 wt%) to silver (2.0 wt%) catalyst promotes the formation of larger silver oxide species.

### 3.3. Methanol oxidation

**Fig. 9:**
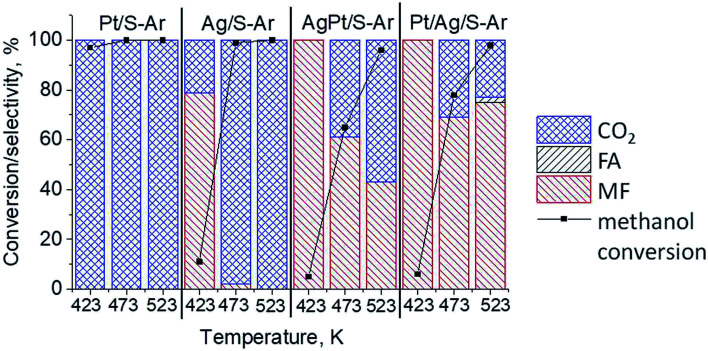
Results of methanol oxidation at 423 K, 473 K and 523 K over the catalysts activated at 673 K in Ar flow (40 mL min^−1^) for 2 h; FA – formaldehyde, MF – methyl formate.

**Fig. 10:**
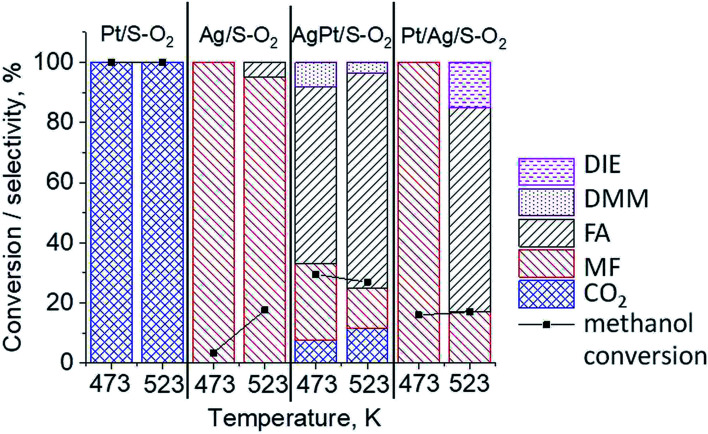
Results of methanol oxidation at 473 K and 523 K over the catalysts activated at 673 K in oxidative mixture flow (10 mL min^−1^ O_2_ + 30 mL min^−1^) for 2 h; DIE – dimethyl ether, DMM – dimethoxymethane, FA – formaldehyde, MF – methyl formate.

**Fig. 11:**
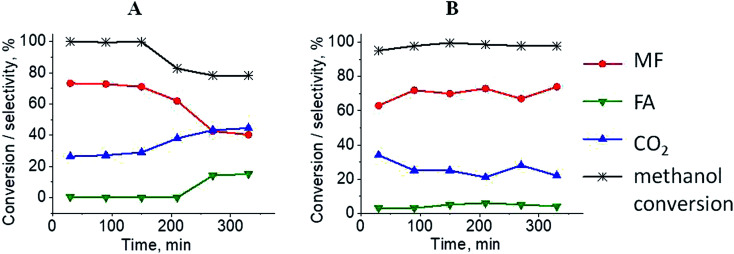
Results of methanol oxidation over 0.5Pt2Ag/M at 423 K (from paper^8^) (A) and Pt/Ag/S at 523 K (B); MF – methyl formate, FA – formaldehyde. Both samples were activated before the reaction for 2 h in ar flow (40 mL min^−1^) at 673 K.

The results of methanol oxidation in the flow system at 423 K, 473 K and 523 K are displayed in Fig. 9. Despite a very low content of platinum, Pt/S–Ar catalyst was very active even below 423 K and led to total oxidation of methanol to carbon dioxide. The other materials were not very active (below 10% of methanol conversion) at 423 K but their activity rapidly increased at 473 K. With increasing methanol conversion over Ag/S–Ar a drastic decrease in selectivity of the reaction was observed and the total oxidation of methanol was the only reaction path at 523 K. Very high activity of Ag/S–Ar sample can be correlated with a large amount of very small Ag NPs (*ca.* 50% of NPs were 1–3 nm) responsible for chemisorption of oxygen and its activity for participation in total oxidation of methanol. Interestingly, although bimetallic materials also ensure high conversion of methanol at 473 K, they appeared to be selective to methyl formate (61% selectivity on AgPt/S–Ar and 69% on Pt/Ag/S–Ar). What is more, the increase in the reaction temperature to 523 K led to 98% conversion of methanol and 75% selectivity to methyl formate when Pt/Ag/S sample was used. These data show that the composition of active species present on the surface of the catalyst prepared by the step-by-step modification favors selective oxidation of methanol to methyl formate. This composition consists of silver oxide, metallic silver and platinum promoter which interacts with silver species. In the sample prepared by modification with silver and platinum using the one-step method the interaction between both metals was weaker, as evidenced by a smaller blue shift in the UV-vis spectra and it resulted in a lower selectivity to methyl formate. It seems that addition of small amounts of platinum (as the promoter) to silver catalyst results in higher resistance of cationic silver (Ag_*n*_^*δ*+^, Ag_2_O) to reduction to metallic species. Unreduced silver species are responsible for high selectivity of bimetallic Ag & Pt catalysts. To check the above mentioned hypothesis, methanol oxidation was performed on the catalysts activated in oxidizing mixture. Such activation enhanced the amount of silver oxide species as shown in Fig. 7 and 8 (described above). As revealed in Fig. 10, the activation of platinum catalyst in oxygen flow did not change the activity and selectivity of this catalyst. However, this activation treatment of Ag/S sample significantly decreased its activity and increased its selectivity (100% and 95% selectivity to methyl formate at 473 and 523 K, respectively) in methanol oxidation. Thus, evidently silver oxide is responsible for activation of the reaction pathway towards methyl formate generation, but it is not responsible for high activity. For higher activity metallic silver and/or platinum are required. Thus, platinum not only acts as the promoter, as mentioned above, but also can provide active centers for activation of adsorbed oxygen. In the absence of silver in the catalyst, platinum catalyzed total oxidation of methanol. Basing on presented results some general conclusion and hypothesis of what is happening in catalysts can be proposed. In combination with silver species, platinum activated the chemisorbed oxygen for further reaction with intermediates chemisorbed on silver oxide. Silver oxide NPs were active centers for chemisorption of formaldehyde (the first product of methanol oxidation chemisorbed on Lewis acid sites as evidenced in ref. 3) and its further reaction with the second methanol molecule towards methyl formate. The increase in the reaction temperature caused easier desorption of formaldehyde and therefore the selectivity to formaldehyde increased and that to methyl formate decreased at 523 K. It was especially pronounced for bimetallic samples (AgPt/S–O_2_ and Pt/Ag/S–O_2_). The question is if doping of silver with a small amount of platinum (shown in this work) would give as efficient catalyst as Ag–Pt alloy containing bimetallic catalysts from our previous studies.^14,17^ For comparison purposes the sample which displayed the best catalytic properties in our previous studies, labelled as 0.5Pt2Ag/M, was taken into account. This sample contained 0.5 wt% of Pt and 1.6 wt% of Ag (Table 1.) which formed bimetallic alloy crystallites loaded on mesoporous silica, MCF type. The yield of methyl formate and the stability of the samples Pt/Ag/S–Ar and 0.5Pt2Ag/M–Ar were considered. As seen in Table 2. Pt/Ag/S–Ar displayed 74% yield of methyl formate at 523 K, whereas the sample from our previous work reached a similar yield at 423 K. Thus, one can conclude that Ag–Pt alloy particles are more active in methyl formate production because the same yield of this product was obtained at a lower temperature. Bearing in mind the easy segregation of Ag–Pt alloy structure, the reaction at 423 K over 0.5Pt2Ag/M–Ar was performed for a longer time and the results are shown in Fig. 11A. After 3 hours of the reaction, a decrease in both, methanol conversion and methyl formate selectivity were noted. Especially significant changes were in the selectivity to methyl formate (decrease from 73% to 40%). The increase in the reaction temperature considerably decreased the methyl formate yield (Table 2). The same test of stability but at 523 K was performed over Pt/Ag/S–Ar catalyst. As shown in Fig. 11B methanol conversion did not change during the reaction time and reached the value of *ca.* 100%. The selectivity to methyl formate was in the range 63–75% and there were no significant changes during the reaction time. The UV-vis spectra of Pt/Ag/S–Ar after stability test, shown in Fig. S4-ESI† confirm the stability of the material and the lack of changes in silver active species. Thus, the Pt/Ag/S catalyst prepared within this work, containing silver doped with platinum species, displays even better catalytic properties than the materials containing Pt & Ag alloy-like nanoparticles. On the one hand, higher temperature of methanol oxidation on Pt/Ag/S–Ar is required (523 K instead of 423 K) for sufficient methyl formate selectivity but on the other hand, very good catalytic properties are preserved for a longer time. What is more, in this study the amount of platinum in catalyst was 10 times lower, which significantly reduced the cost of the material.

**Table 2:** The selectivity and yield of methyl formate (MF) in methanol oxidation over Pt/Ag/S and 0.5Pt2AgPt/M (from previous studies^8^). Both samples were activated at 673 K in Ar flow (40 mL min^−1^), for 2 h before the reaction

Catalyst	Temp., K	Methanol conv., %	Selectivity to MF	Yield of MF
Pt/Ag/S–Ar	423	6	100	6.0
	473	78	69	54
	523	98	75	74
0.5Pt2Ag/M–Ar^a^	373	100	59	59
	423	100	73	73
	473	74	17	13

a Sample from previous work.^8^

## 4. Conclusions

The results presented in this work not only allow construction of effective bimetallic (Ag & Pt) catalyst for production of methyl formate from methanol but also contributed to better understanding of the role of each component in this bimetallic catalyst in activity and selectivity in methanol oxidation. Metallic silver Ag^0^ was found to be responsible for chemisorption of oxygen, while the platinum active phase took part in activation of adsorbed oxygen, too. Platinum species also acted as an electronic promoter and preserved total reduction of silver oxide upon activation and reaction conditions. This role was crucial because silver oxide was responsible for selective formation of methyl formate (*via* chemisorption of formaldehyde on Lewis acid sites). Thus, addition of small amount of platinum (as the promoter) to silver catalyst resulted in a higher resistance of cationic silver (Ag_*n*_^*δ*+^, Ag_2_O) to reduction and increased the activity and selectivity in oxidation of methanol to methyl formate. Interaction between platinum promoter and silver species ensured the best catalytic properties (activity, selectivity, stability) and such properties were observed for the sample prepared by the step-by-step method (Pt/Ag/S). The catalyst obtained in this method displayed 74% yield of methyl formate production at 523 K and it was stable in the reaction time.

## Conflicts of interest

There are no conflicts to declare.

## Supplementary Material

RA-010-D0RA01562H-s001
