# Supplementary material for: A platinum promoted Ag/SBA-15 catalyst effective in selective oxidation of methanol – design and surface characterization
Source: RSC Adv. 2020 Apr 9;10(25):14570–80. doi: 10.1039/d0ra01562h (PMC9051898; doi:10.1039/d0ra01562h)
Supplement: RA-010-D0RA01562H-s001 [file RA-010-D0RA01562H-s001.pdf]

Electronic Supplementary Material (ESI) for RSC Advances.
This journal is © The Royal Society of Chemistry 2020

Platinum promoted Ag/SBA-15 catalyst effective in selective oxidation of methanol – design and surface characterization

Joanna Wisniewska*, Izabela Dziedzic, Maria Ziolek

Adam Mickiewicz University, Poznań, Faculty of Chemistry, Uniwersytetu Poznańskiego 8, 61-614 Poznań, Poland

S1

Functionalization of SBA-15 support with 3-mercaptopropyltrimethoxysilane (MPTMS) [1]

Support was grafted with 3-mercaptopropyltrimethoxysilane (MPTMS) (95%, Sigma-Aldrich) to functionalize its surface before modification with metals. 7 g of the support powder was refluxed in a dry toluene solution (200 mL) containing 18.9 mL MPTMS at 373 K for 18 h. The reagents were not purified before use. The material was recovered by filtration followed by washing in: dry toluene (200 mL), ethanol (200 mL) and distilled water (100 mL). The powder was dried in an oven at 353 K.

S2

Samples characterization [1][2]

ICP-OES

The inductively coupled plasma optical emission spectrometer (ICP-OES) ICPE-9820 (Shimadzu, Japan) with a mini-torch was used for qualitative and quantitative detection of selected elements in studied samples. Before the analysis, about 100 mg of a sample studied and 4 mL H_2O, 3 mL H_2SO_4, 3 mL H_3PO_4 and 2 mL HF (all reagents were for trace analysis, Sigma Aldrich, USA) were added to a teflon tube and mineralized in Microwave oven (Anton Paar, Austria) in three steps: ramp time 5 min. (1500 W), hold time 50 min. (1500 W) and cooling to 343 K.

X-ray diffraction (XRD)

XRD measurements were carried out on a Bruker AXS D8Advance diffractometer with Cu K_α radiation (λ= 0.154 nm), with a step size of 0.05° in the small-angle range (1°–10°) and with a step size of 0.2° in the wide-angle range (6°–60°).

N_2 adsorption/desorption isotherms

The N_2 adsorption-desorption isotherms were obtained at 77 K using a Micromeritics ASAP 2020 Physisorption Analyzer. Before measurements, the samples were degassed at 573 K for 8 h. The surface area was calculated by the BET method, whereas the volume and diameter of pores were determined by the DFT method.

UV–vis spectroscopy

UV–vis spectra were recorded using a Varian-Cary 300 Scan UV–visible spectrophotometer. Powder samples were placed in a cell equipped with a quartz window. The spectra were recorded in the range from 800 to 190 nm. Spectralon was used as the reference material.

X-ray Photoelectron Spectroscopy (XPS)

X-ray Photoelectron Spectroscopy (XPS) was performed on an Ultra-high vacuum photoelectron spectrometer based on Phoibos150 NAP analyzer (Specs, Germany). The analysis chamber was operated under vacuum with a pressure close to 5×10^{-9} mbar and the sample was irradiated with a monochromatic Al $_{K\alpha}$ (1486.6 eV) radiation (15 kV; 10 mA). Spectra were recorded with a flood gun acting as neutralizer. Binding energies were referenced to the Si 2p peak from silica 103.4 eV

Transmission electron microscopy (TEM)

For transmission electron microscopy (TEM) measurements, the powders were deposited on a grid covered with a holey carbon film and transferred to JEOL 2000 electron microscope operating at 80 kV. Samples were made in the form of suspension in 1-butanol.

References

[1] J. Czaplinska, P. Decyk, M. Ziolek, Surface properties and catalytic performance of Pt–Ag supported on silica – The effect of preparation methods, Appl. Catal. A Gen. 504 (2015) 361–372. doi:10.1016/j.apcata.2014.12.054.

[2] J. Wisniewska, M. Ziolek, N. Artioli, M. Daturi, The effect of niobium and tantalum on physicochemical and catalytic properties of silver and platinum catalysts based on MCF mesoporous cellular foams, J. Catal. 336 (2016) 58–74. doi:10.1016/j.jcat.2015.12.018.

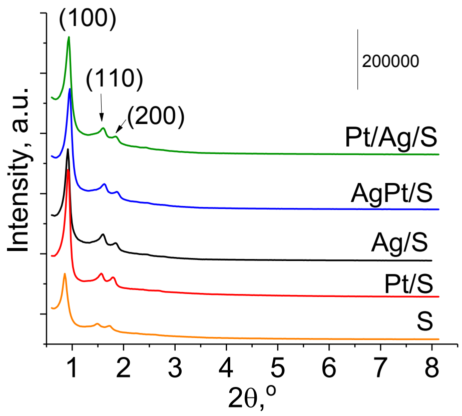

Figure S1. Low-angle XRD patterns of SBA-15 (denoted S) and the support modified with platinum and/or silver.

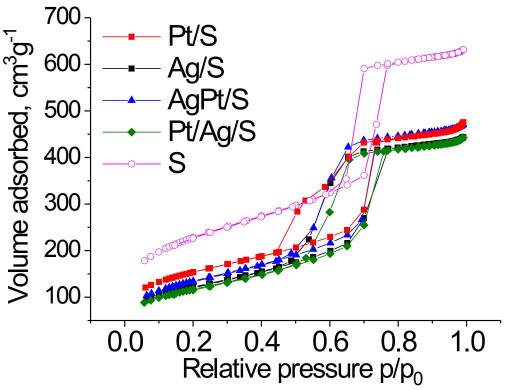

Figure S2. Nitrogen adsorption/desorption isotherms of the samples calcined at 773 K for 4 h.

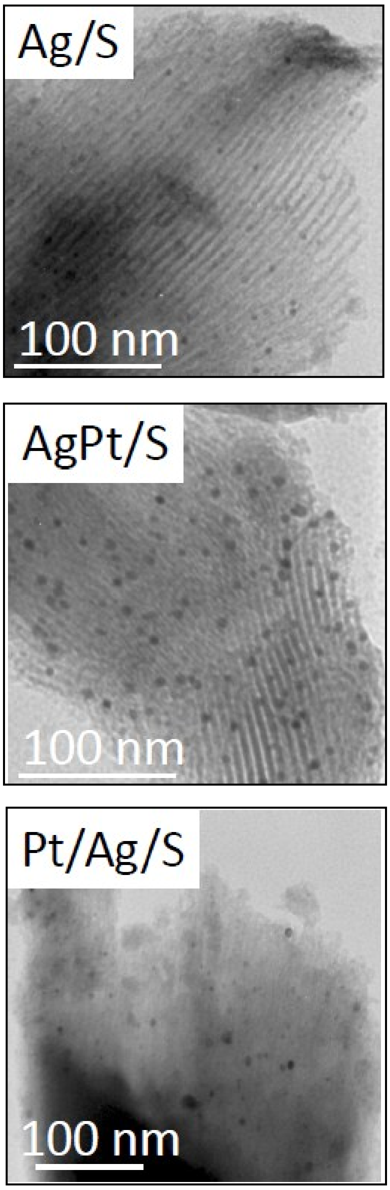

Figure S3. TEM images of the samples activated at 673 K in argon flow (40 mL/min) for 2 h.

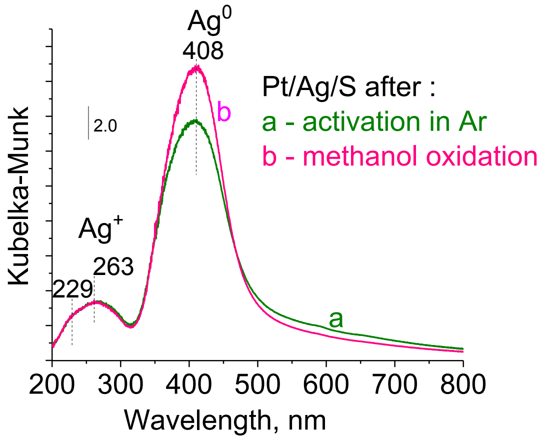

Figure S4. UV-vis spectra of Pt/Ag/S catalyst: activated at 673 K in Ar flow (40 mL/min) for 2 h (a) and after methanol oxidation performed at 523 K for 330 min (b)
